# Recent advances in high‐resolution traveling wave‐based ion mobility separations coupled to mass spectrometry

**DOI:** 10.1002/mas.21902

**Published:** 2024-08-01

**Authors:** Cameron N. Naylor, Gabe Nagy

**Affiliations:** ^1^ Department of Chemistry University of Utah Salt Lake City Utah USA

**Keywords:** ion mobility spectrometry, isomers, mass spectrometry, separation science

## Abstract

Recently, ion mobility spectrometry‐mass spectrometry (IMS‐MS) has become more readily incorporated into various omics‐based workflows. These growing applications are due to developments in instrumentation within the last decade that have enabled higher‐resolution ion mobility separations. Two such platforms are the cyclic (cIMS) and structures for lossless ion manipulations (SLIM), both of which use traveling wave ion mobility spectrometry (TWIMS). High‐resolution separations achieved with these techniques stem from the drastically increased pathlengths, on the order of 10 s of meters to >1 km, in both cIMS‐MS and SLIM IMS‐MS, respectively. Herein, we highlight recent developments and advances, for the period 2019–2023, in high‐resolution traveling wave‐based IMS‐MS through instrumentation, calibration strategies, hyphenated techniques, and applications. Specifically, we will discuss applications including CCS calculations in multipass IMS‐MS separations, coupling of IMS‐MS with chromatography, imaging, and cryogenic infrared spectroscopy, and isomeric separations of glycans, lipids, and other small metabolites.

**Abbreviations**
CCS: ion‐neutral collisional cross section
CID: collisional induced dissociation
cIMS: cyclic ion mobility spectrometry
CRIMP: compression ratio ion mobility programing
cryo‐IR: cryogenic infrared spectroscopy
DC: direct current
DIY: do‐it‐yourself
GC: gas chromatography
HRTWIMS: high resolution traveling wave ion mobility spectrometry
IgG#: immunoglobin
IMS‐MS: ion mobility spectrometry‐mass spectrometry
IMS^n^
: multi‐dimensional ion mobility spectrometry
LC: liquid chromatography
LC‐MS: liquid chromatography‐mass spectrometry
LESA: liquid extraction surface analysis
PC: phosphatidylcholine
PCB: printed circuit board
PFAS: perfluorinated alkyl substances
PNNL: Pacific Northwest National Laboratory
RF: radio frequency
RNA: ribonucleic acid
SLIM: structures for lossless ion manipulations
SMILES: simplified molecular input line entry system
SUPER: serpentine ultralong path with extended routing
TOF: time of flight
TWIMS: traveling wave ion mobility spectrometry

## 1 INTRODUCTION/INSTRUMENTATION

Ion mobility spectrometry‐mass spectrometry (IMS‐MS) is an analytical technique where ions are separated on a millisecond timescale in the gas phase based on their size, shape, and charge (i.e., their ion mobility) under the presence of an electric field. Strategies to improve resolution in IMS‐MS are like other separations, where an increased separation pathlength is necessary. Historically, IMS‐MS has been restricted to drift tubes, where a static electric field is applied to a fixed length (Eiceman et al., 2014); however, improving resolution in drift tubes is limited by the required voltage drop for an extended pathlength. Notable efforts to improve IMS‐MS resolution were made by Clemmer's group in the 2000s, where they designed 2 (Koeniger et al., 2006) and 3 (Merenbloom et al., 2006, 2008) meter‐long tandem drift tube IMS‐MS instruments. Unfortunately, physical limitations make it impractical to improve resolution further. Therefore, other approaches are needed to push high‐resolution IMS‐MS techniques to the next generation. The Clemmer group's solution to increasing the pathlength was the cyclotron drift tube, which is constructed with eight segments: four curved drift tubes connected by four ion funnel transmission regions (Merenbloom et al., 2009). This variable pathlength instrument enabled IMS resolving powers >1000 to be achieved (Glaskin et al., 2013).

**Figure 1:**
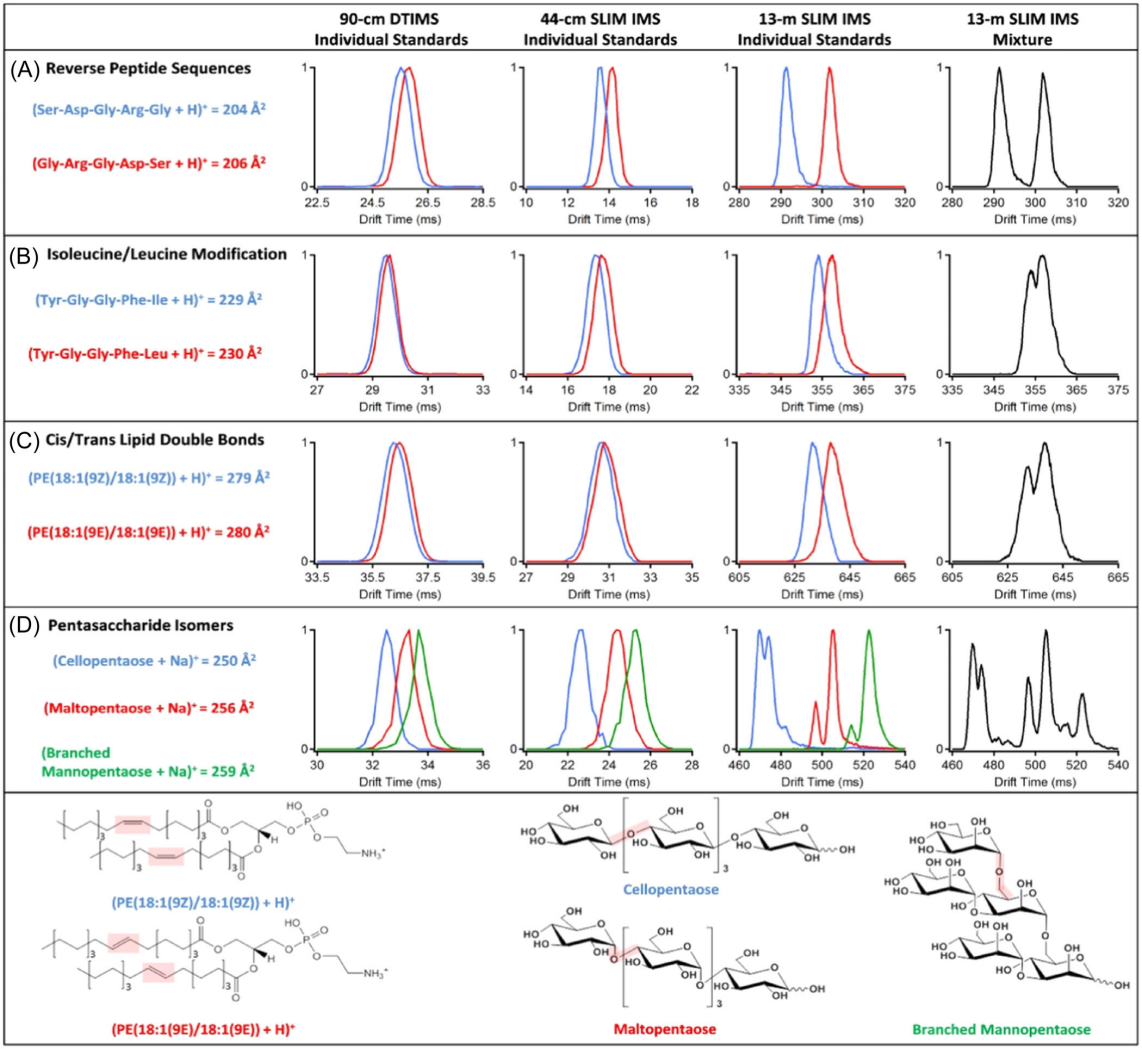
Comparison of isomeric resolution between a 90 cm drift tube ion mobility spectrometry‐mass spectrometry (IMS‐MS), 44 cm structures for lossless ion manipulations (SLIM) IMS‐MS, and 13 m SLIM IMS‐MS platform for reverse peptide sequences (A), isoleucine/leucine modifications (B), cis/trans double bonds (C), and pentasaccharides (D). Reprinted (adapted) with permission from Deng L, Ibrahim YM, Baker ES, Aly NA, Hamid AM, Zhang X, Zheng X, Garimella SVB, Webb IK, Prost SA, Sandoval JA, Norheim RV, Anderson GA, Tolmachev AV, Smith RD. 2016. Ion mobility separations of isomers based upon long path length structures for lossless ion manipulations combined with mass spectrometry. ChemistrySelect. 1:2396–2399. Copyright 2016 John Wiley & Sons Inc. [Color figure can be viewed at wileyonlinelibrary.com]

In 2004, the traveling wave ion mobility spectrometry platform (TWIMS) was developed by Giles et al. (2004), which operates by pulsing DC traveling waves in the separation region with velocities ~100 s of m/s and amplitudes 10 s of V. The commercial version of this instrument, sold by Waters as the Synapt, has a defined pathlength of 25 cm, which also limits achievable resolution. In 2014, Pacific Northwest National Labs (PNNL) premiered a new longer pathlength traveling wave‐based ion mobility spectrometry platform called structures for lossless ion manipulations (SLIM) (Garimella et al., 2014; Tolmachev et al., 2014; Webb et al., 2014). SLIM IMS‐MS separations occur between two mirrored printed circuit boards containing the TW, RF, and DC voltages, which affords flexibility to users in designing boards with specific capabilities. SLIM IMS‐MS designs quickly developed, and two notable variants debuted in 2017: a 13.5‐m single pass pathlength with the ability to cycle ions extended pathlengths to 100 s of meters (serpentine ultralong path with extended routing, SUPER) (Deng et al., 2017) and a design that enabled ion compression to maximize ion populations that could be sampled (compression ratio ion mobility programming, CRIMP) (Deng et al., 2017). Both capabilities will be discussed in detail in a later section. These increased pathlengths enabled higher resolution separations than had been achieved with drift tube IMS‐MS systems (Figure 1) (Deng et al., 2016). In 2021, a commercialized SLIM IMS‐MS platform was released from MOBILion Systems.

**Figure 2:**
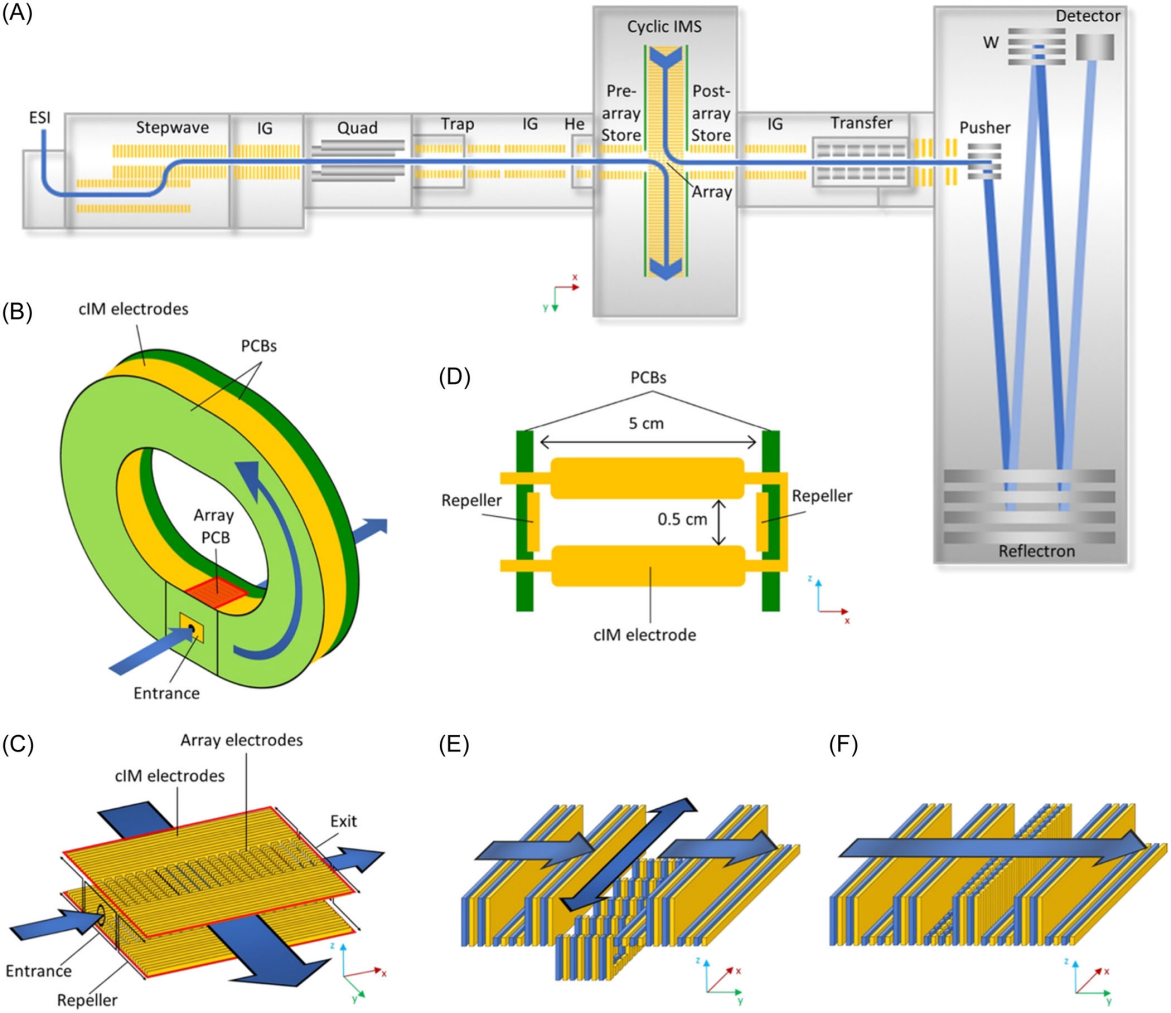
Diagram of the cyclic ion mobility spectrometry‐mass spectrometry (cIMS‐MS) instrument (A). Diagram of the cIMS array (B) highlighting how ions are separated orthogonally to their entrance (C). Diagram of the PCB arrangement in the cIMS separation region (D). Diagram highlighting the directionality of the traveling waves during separation (E) and ejection to the TOF‐MS for detection (F). Reprinted (adapted) with permission from Giles K, Ujma J, Wildgoose J, Pringle S, Richardson K, Langridge D, Green M. 2019. A Cyclic Ion Mobility‐Mass Spectrometry System. Anal Chem. 91:8564–8573. Copyright 2019 American Chemical Society. [Color figure can be viewed at wileyonlinelibrary.com]

While PNNL was refining their SLIM IMS‐MS designs, Waters developed their own solution to limited pathlengths with the cyclic ion mobility spectrometry‐mass spectrometry (cIMS‐MS) platform (Giles et al., 2019), which was inspired by the Clemmer group's cyclotron drift tube. Commercially launched in 2019, the cIMS‐MS is a TWIMS variant with a 1 m single pass separation region (Figure 2), and as in SLIM SUPER, ions can be cycled multiple passes thus enabling high‐resolution IMS‐MS separations. When comparing the resolving powers of these various platforms, SLIM IMS‐MS can achieve >1500 after 1 km (Deng et al., 2017), while cIMS‐MS can achieve >800 after 100 m (Giles et al., 2019), both of which greatly exceed the 1 m Agilent DTIMS (Rp ~50–60) (May et al., 2015) and the 25 cm Waters TW Synapt (Rp ~30–40) (Giles et al., 2011).

Due to the recent commercialization and improving instrumentation technology, there has been a growing number of publications that use these two high‐resolution TWIMS platforms (i.e., the SLIM IMS‐MS and cIMS‐IMS) within the past few years. In this review, for the period 2019 to 2023, we will cover advances they have enabled. We anticipate that as these techniques become more widely adopted and integrated into existing analytical workflows, they will enable tremendous advances in omics‐based research.

### 1.1 Recent advances in structures for lossless ion manipulations (SLIM) designs

**Figure 3:**
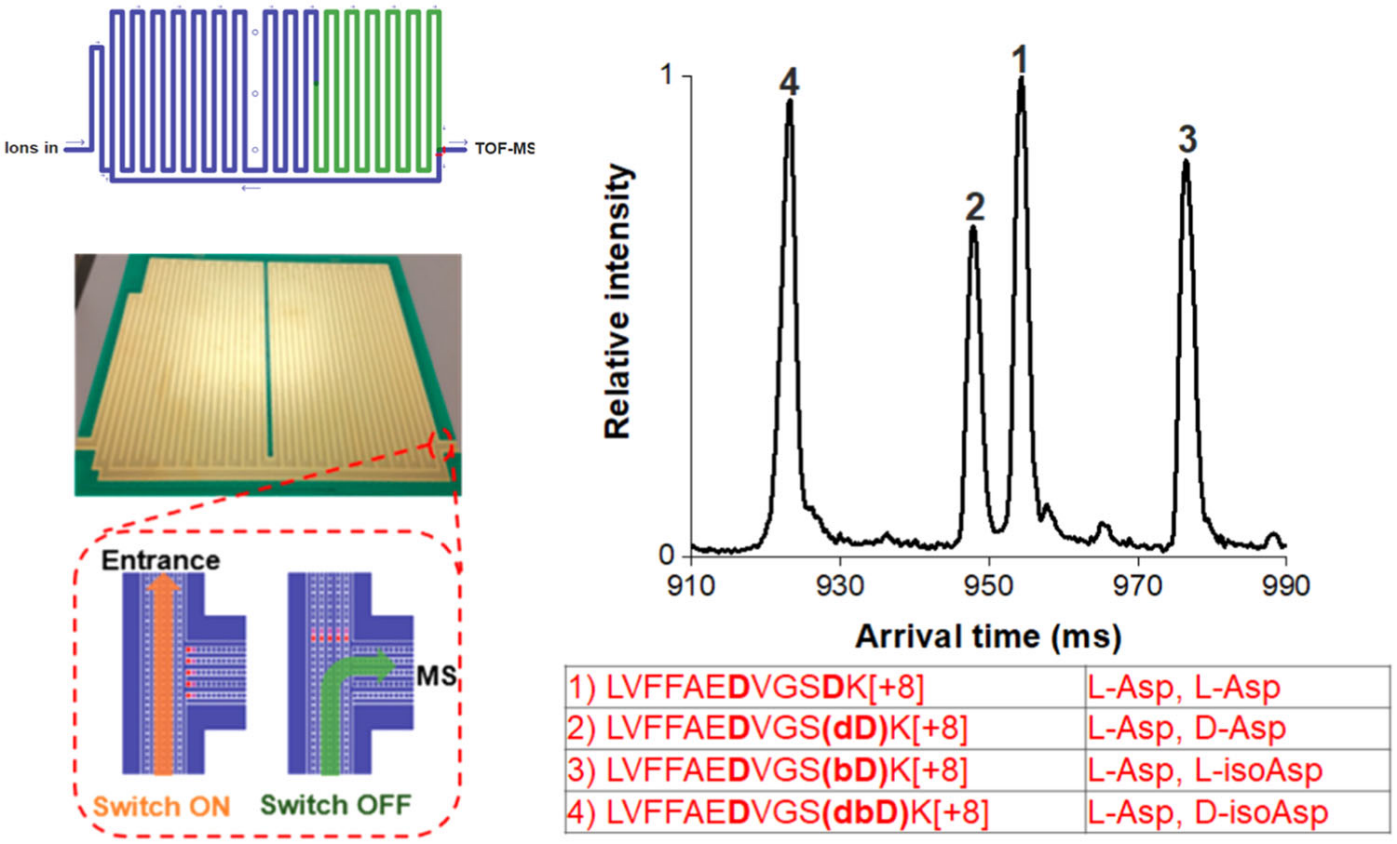
Diagram of the structures for lossless ion manipulations (SLIM) serpentine ultralong path with extended routing (SUPER) ion mobility spectrometry‐mass spectrometry (IMS‐MS) platform and a picture of one PCB (left). 72 m SLIM SUPER IMS‐MS separation of four beta‐amyloid peptide epimers (right). Reprinted (adapted) with permission from Deng L, Webb IK, Garimella SVB, Hamid AM, Zheng X, Norheim RV, Prost SA, Anderson GA, Sandoval JA, Baker ES, Ibrahim YM, Smith RD. 2017. Serpentine Ultralong Path with Extended Routing (SUPER) High‐Resolution Traveling Wave Ion Mobility‐MS using Structures for Lossless Ion Manipulations. Anal Chem. 89:4628–4634. Copyright 2017 American Chemical Society. Reprinted (adapted) Nagy G, Kedia K, Attah IK, Garimella SVB, Ibrahim YM, Petyuk VA, Smith RD. 2019. Separation of β‐amyloid tryptic peptide species with isomerized and racemized l‐aspartic residues with ion mobility in structures for lossless ion manipulations. Anal Chem. 91:4374–4380. with permission from Copyright 2019 American Chemical Society. [Color figure can be viewed at wileyonlinelibrary.com]

**Figure 4:**
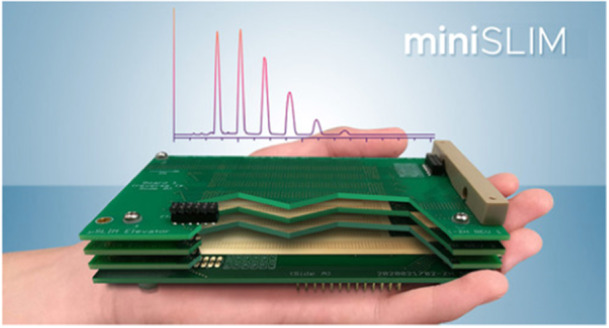
Picture of the miniSLIM platform highlighting its compact instrument footprint. Reprinted (adapted) with permission from Hollerbach AL, Norheim RV, Kwantwi‐Barima P, Smith RD, Ibrahim YM. 2022. A miniature multilevel structures for lossless ion manipulations ion mobility spectrometer with wide mobility range separation capabilities. Anal Chem. 94:2180–2188. Copyright 2022 American Chemical Society. [Color figure can be viewed at wileyonlinelibrary.com]

As mentioned earlier, the use of traveling wave ion mobility separations is necessary to achieve higher resolution. The greatest technological advancement related to achieving higher resolution, from extended pathlength separations, stemmed from the ability to route ions around 90° turns in SLIM‐based designs (Garimella et al., 2014; Tolmachev et al., 2014; Webb et al., 2014). This led to the development of a 13.5‐m pathlength SLIM IMS‐MS platform, which greatly outperformed 1‐m DTIMS platforms in terms of isomer separations (Deng et al., 2017). To further increase achievable resolution, a switch that enables ions to either be routed to the TOF for MS detection or to the beginning of the serpentine path was developed. This development, termed serpentine ultralong path with extended routing (SUPER), allowed SLIM IMS‐MS separations on the order of 100 s of meters and enabled the separation of challenging isomeric species that were previously intractable with other platforms (Figure 3). However, these ultralong pathlength separations suffered from poor sensitivity related to the number of ions initially accumulated and released via the ion funnel trap. This led to the development of in‐SLIM ion accumulation, which greatly increases the total ion population generated as compared to that in an ion funnel trap (Garimella, Hamid, et al., 2016; Garimella, Ibrahim, et al., 2016). It was demonstrated that 5 × 10^9^ charges could be introduced with in‐SLIM ion accumulation, resulting in 2 orders of magnitude increase in charge capacity compared to that from an ion funnel trap. In‐SLIM ion accumulation is performed by accumulating ions in one traveling wave region using a gentle traveling wave amplitude and frequency while keeping a second traveling wave halted to create a DC wall. With these larger ion populations generated from in‐SLIM ion accumulation comes a larger magnitude gate width contribution which would also decrease achievable IMS‐MS resolution at longer separation pathlengths. This led to the development of compression ratio ion mobility programming (i.e., CRIMP), where ions are spatially compressed at the interface of two traveling waves using a stuttering (i.e., intermittently moving) TW in the second region (Garimella, Hamid, et al., 2016). The use of CRIMP in conjunction with in‐SLIM ion accumulation and SLIM SUPER IMS‐MS separations greatly improved both resolution and sensitivity of measurements (Deng et al., 2017). In an effort to improve the overall peak capacity, ion elevators and escalators were implemented across four levels in SLIM IMS‐MS separations (Hollerbach et al., 2020). As shown in Figure 4, another notable recent advance in SLIM design and geometry includes the miniSLIM: a 1‐m separation pathlength performed in a highly compact footprint (Hollerbach et al., 2022). To improve the sensitivity of low abundance species in SLIM IMS‐MS measurements, a high charge capacity array of traps design was developed (Attah et al., 2019; Kwantwi‐Barima et al., 2023). This array of traps enabled low‐intensity species to be accumulated and subsequently released for later separation, thus improving the overall signal‐to‐noise and sensitivity. The flexibility of the SLIM platform was recently demonstrated by Deng et al. where a dual‐track SLIM was implemented as an ion filter coupled to a triple‐quadrupole mass spectrometer (Deng et al., 2022). While IMS separations are most often coupled with TOF mass analyzers because of their speed, a dual gate approach was used to couple SLIM IMS separations with an Orbitrap mass analyzer (Hollerbach et al., 2023). This enabled high resolution in both the IMS and MS dimensions, and we envision that this approach will become more mainstream.

In addition to the efforts at PNNL over the past decade, and the subsequent commercialization of SLIM IMS‐MS by MOBILion Systems, a few research groups have developed their own SLIM designs. Such examples of this include: Rizzo's group which coupled SLIM IMS‐MS with cryogenic infrared spectroscopy, Bush's group modular SLIM IMS‐MS designs (Eaton et al., 2019, 2023; Zercher et al., 2023), and Clowers' group DIY SLIM IMS‐MS initiative (Kinlein et al., 2022) with open‐source designs freely available (Greer et al., 2023).

## 2 APPLICATIONS

### 2.1 Collision cross sections (CCS)

In IMS‐MS techniques that use a nonstatic electric field, calibration procedures must be used to obtain an ion's reduced mobility (K_0_) or rotationally averaged ion‐neutral CCS. Traditionally, TWIMS calibration procedures, both in SLIM IMS‐MS and cIMS‐MS, use a power function that correlates arrival times to mobilities or CCS (Ruotolo et al., 2008). Other notable approaches include the use of a blend function to account for velocity relaxation in larger molecular weight ions (Richardson et al., 2021) as well as the use of average ion velocities to enable pathlength independent CCS measurements in multipass IMS‐MS separations (Habibi & Nagy, 2023a).

Regarding calibrant selection in TWIMS, it has been demonstrated that matching the molecule class between calibrant and unknown will enable better accuracy (Hines et al., 2016). This potential for molecular class biases in CCS measurements has also been investigated in high‐resolution TWIMS‐MS separations (Li et al., 2020; Rose et al., 2022). Ultimately, it was concluded that the use of rigid phosphazene calibrants in conjunction with a scaling factor resulted in both improved precision and accuracy in TWIMS CCS across varying molecular classes (Rose et al., 2022). Another factor to consider in calibrant selection is the potential for unwanted ion heating to occur that may bias CCS toward more unfolded structures, particularly for peptides and proteins (Li et al., 2020; Ruotolo et al., 2008).

**Figure 5:**
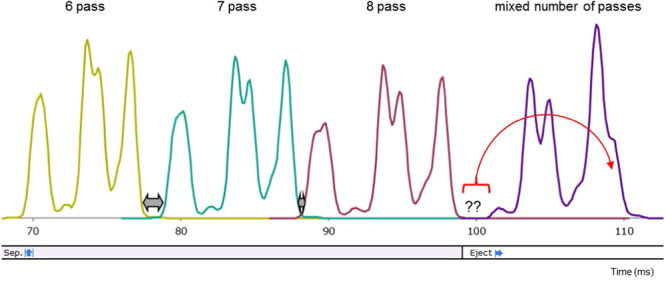
Depiction of the wrap‐around effect in cyclic ion mobility spectrometry‐mass spectrometry, where faster ions (red “??” annotation) lap slower ions. Reprinted (adapted) with permission from Giles K, Ujma J, Wildgoose J, Pringle S, Richardson K, Langridge D, Green M. 2019. A cyclic ion mobility‐mass spectrometry system. Anal Chem. 91:8564–8573. Copyright 2019 American Chemical Society. [Color figure can be viewed at wileyonlinelibrary.com]

Another challenge in HRTWIMS calibration is the phenomenon of ion lapping or the wrap‐around effect, where faster, higher‐mobility, ions will lap slower, lower mobility, ones as shown in Figure 5 (Giles et al., 2019). This effect makes it difficult to perform CCS calibration because ions of interest may have been subjected to a different number of passes thus making arrival time irrelevant. Habibi and Nagy addressed this issue developing a calibration procedure that uses average ion velocities rather than absolute arrival times (Habibi & Nagy, 2023a). This procedure enables CCS to be calculated in a pathlength‐independent manner and can be applied to high‐resolution separations in both cIMS and SLIM.

#### 2.1.1 Varying the traveling wave profile

While square waves are most common in TWIMS, previous studies have explored the use of alternative waveforms and their effect on CCS. Conant et al. and Kwantwi‐Barima et al. explored how four different waveforms, sine, square, triangle, and sawtooth, affect resolution, sensitivity, and accuracy of CCS in SLIM IMS‐MS (Conant et al., 2021; Kwantwi‐Barima et al., 2022). They concluded that for select applications other waveforms may outperform the commonly used square one in TWIMS. A follow‐up of this work was from Li et al. which used SIMION to simulate the effects of different waveforms in SLIM IMS‐MS (Li et al., 2019). Kinlein and Clowers explored the use of dynamic traveling waves (i.e., waveforms that are ramped or changed in the experiment to increase the amplitude or decrease the frequency), where they found improved resolution and sensitivity could be observed with select conditions. Similarly, Williamson and Nagy developed a temporal compression technique by ramping the traveling wave amplitude at the end of a separation, which resulted in an increase in intensity and respective decrease in a peak's full width half maximum by a factor of four (Williamson & Nagy, 2022a). One final effort that improved both sensitivity and the signal‐to‐noise ratio in SLIM IMS‐MS measurements was where Clowers et al. implemented Hadamard multiplexing (Clowers et al., 2021). Future work is needed to explore if alternative waveforms could help in minimizing ion heating in TWIMS separations and their potential for improving resolution in longer separation pathlengths.

### 2.2 Isotopic shifts

**Figure 6:**
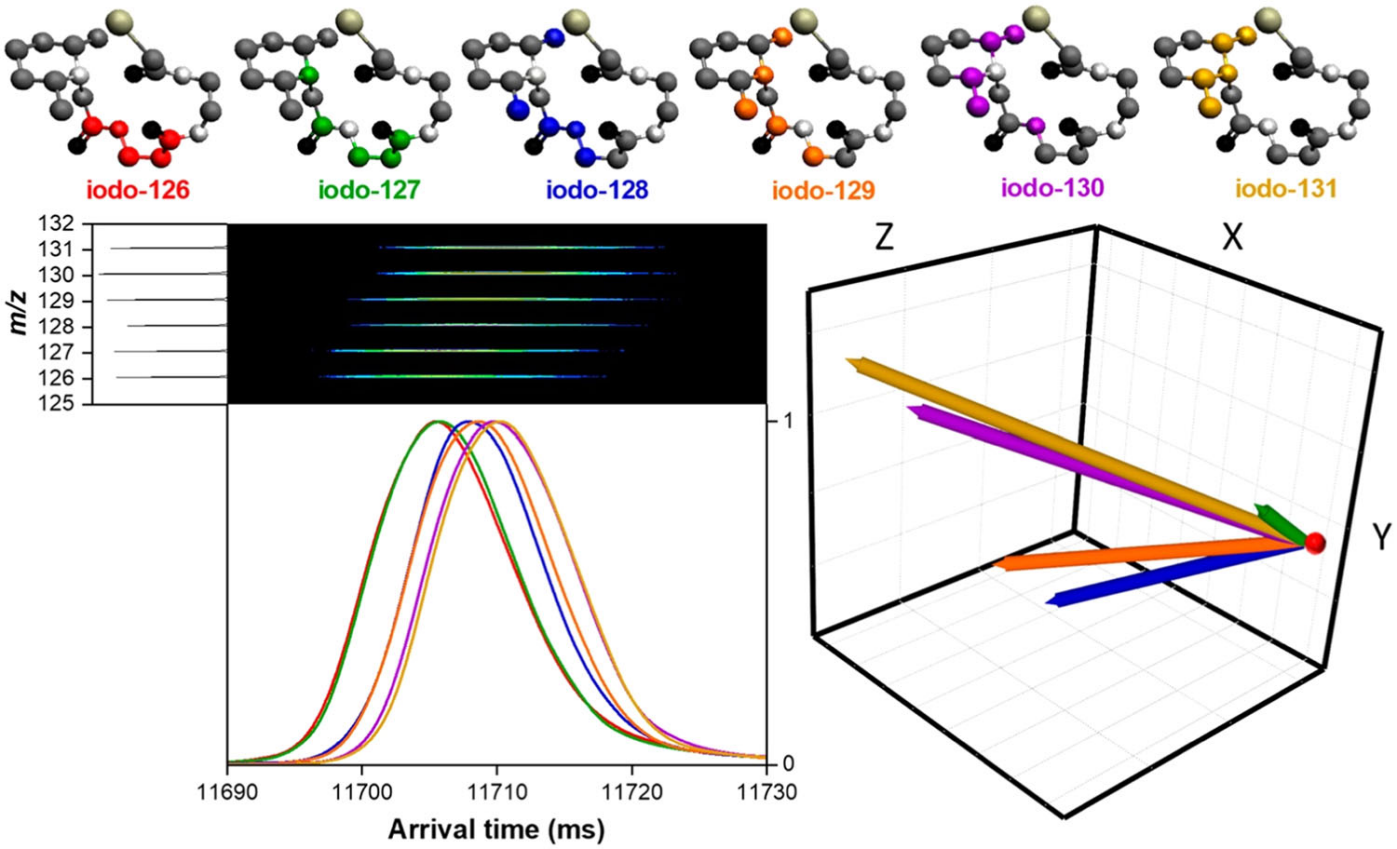
A 1354.5 m structures for lossless ion manipulations (SLIM) serpentine ultralong path with extended routing ion mobility spectrometry‐mass spectrometry separation of six isotopomers based on their respective mass distributions from their isotopic substitutions. Reprinted (adapted) with permission from Wojcik R, Nagy G, Attah IK, Webb IK, Garimella SVB, Weitz KK, Hollerbach A, Monroe ME, Ligare MR, Nielson FF, Norheim RV, Renslow RS, Metz TO, Ibrahim YM, Smith RD. 2019. SLIM ultrahigh resolution ion mobility spectrometry separations of isotopologues and isotopomers reveal mobility shifts due to mass distribution changes. Anal Chem. 91:11952–11962. Copyright 2019 American Chemical Society. [Color figure can be viewed at wileyonlinelibrary.com]

An article from Valentine and Clemmer speculated that given high enough IMS‐MS resolution, isotopologues, molecules that differ in their number of neutrons, could be resolved based on differences in their respective reduced mass terms (Valentine & Clemmer, 2009). In a study done on a SLIM SUPER IMS‐MS platform (Wojcik et al., 2019), the isotopologues of various small molecules were resolved after >1 km of separation (Figure 6). In that work, it was noted that the peak widths of subsequent isotopologues were wider than that of [M], potentially from the presence of a mixture of isotopomers (i.e., isotopic isomers) in each individual isotopologue. From there, based on a theoretical mobility calibration trendline, it was shown that certain heavy‐labeled arginine isotopologues fell off the trendline indicating that other physical contributions such as those from mass distribution could be at play. This led to the SLIM SUPER IMS‐MS separations of tandem mass tag isotopomers, which stemmed from differences in their respective mass distributions (i.e., changes in center of mass and moments of inertia) (Harrilal et al., 2023; Wojcik et al., 2019). In addition to this study, a follow‐up one showed great agreement between theoretical isotopic shifts and experimental ones through the IMoS 2.0 software from the Larriba‐Andaluz group (Harrilal et al., 2021). Williamson and Nagy demonstrated experimental estimated relative mobility differences amongst isotopologue and isotopomers with cIMS‐MS (Williamson & Nagy, 2022b, 2023; Williamson & Trimble, & Nagy, 2022, 2023). From there, they demonstrated isomer and conformer‐specific isotopic shifts based on mass distribution. Recently, they have utilized derivatization strategies (e.g., hydrogen‐deuterium exchange and deuterated permethylation) to introduce isotopic shifts, which have been shown to be orthogonal to CCS (Williamson & Nagy, 2023; Williamson & Trimble, & Nagy, 2023). Future computational work (e.g., using IMoS 2.0) is needed to better understand and deconvolute the center of mass and moments of inertia contributions in these mass distribution‐based isotopic shifts.

### 2.3 Hyphenated techniques

With high‐resolution, multipass, systems, the consideration of experimental timescales becomes paramount in the coupling of IMS to other techniques. Specifically, the IMS separation must still be fast enough to ensure sufficient peak sampling to maintain integrity of the overall peak shape from any prior front‐end technique/separation. As outlined below, a handful of studies have recently been published coupling HRTWIMS with other techniques.

#### 2.3.1 Coupling liquid chromatography with high‐resolution ion mobility separations

**Figure 7:**
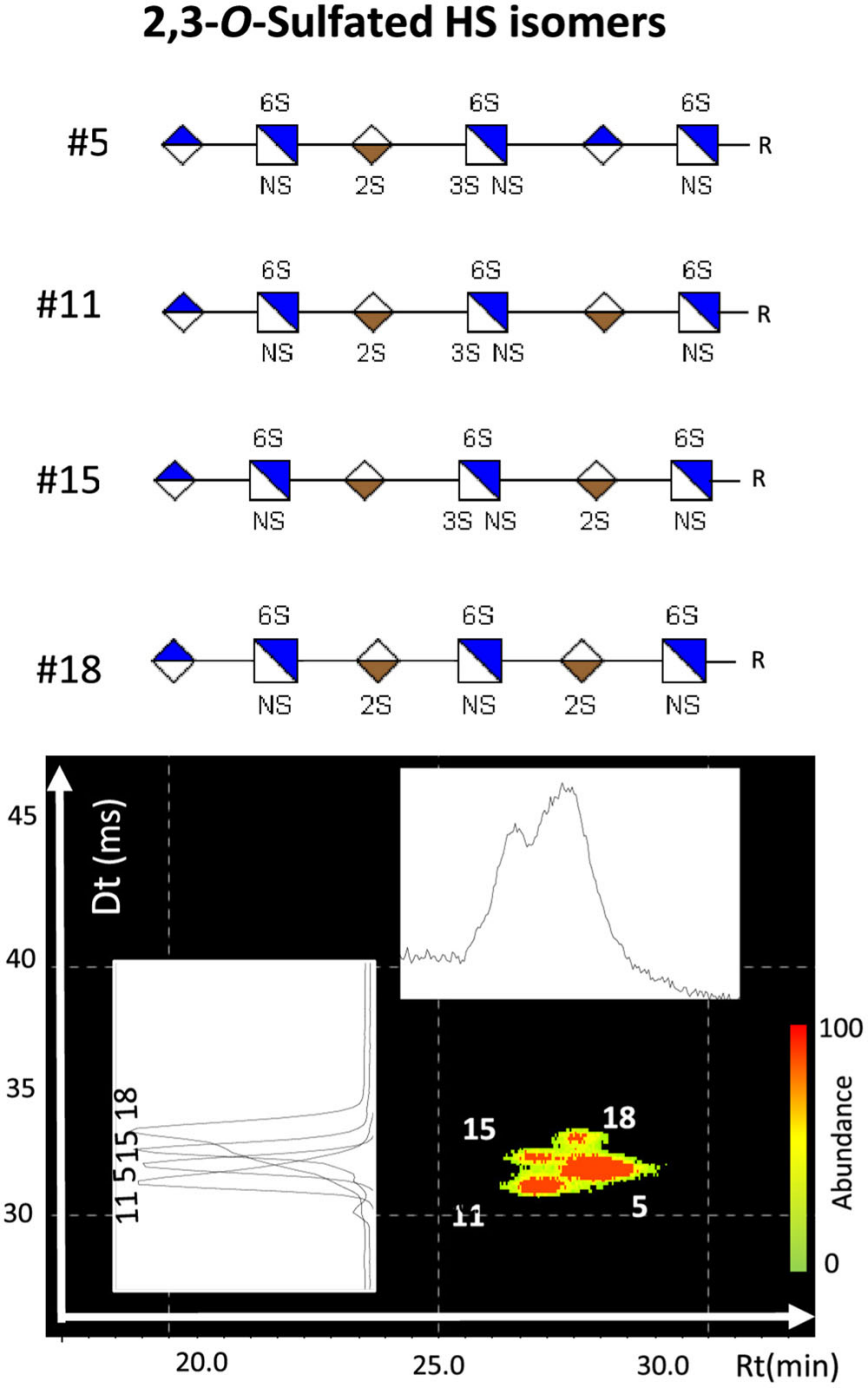
Improved peak capacity in the separation of heparan sulfate oligosaccharide isomers when coupling liquid chromatography with cyclic ion mobility spectrometry‐mass spectrometry. Reprinted (adapted) with permission from Cavallero GJ, Zaia J. 2022. Resolving heparan sulfate oligosaccharide positional isomers using hydrophilic interaction liquid chromatography‐cyclic ion mobility mass spectrometry. Anal Chem. 94:2366–2374. Copyright 2022 American Chemical Society. [Color figure can be viewed at wileyonlinelibrary.com]

Both SLIM IMS‐MS and cIMS‐MS have been coupled with liquid chromatography to improve the overall peak capacity in the analyses of complex mixtures. Pesticide isomers combined with metal adduction, heparan sulfate oligosaccharides, and isobaric and racemic mixtures of peptides, are recent examples of 2D LC‐IMS‐MS separations (Cavallero & Zaia, 2022; McCullagh et al., 2021; Tomczyk et al., 2021). An example of improved peak capacity was demonstrated by Cavallero and Zaia, where two separate peaks are shown in the LC and IMS domains, but combined, four total heparan sulfate oligosaccharide isomers are present (Figure 7). SLIM IMS‐MS was coupled to LC to separate and identify phosphatidylcholine isomers that differ by double bond location in total brain and liver extracts (Kedia et al., 2023). Other examples include applications in proteomics (Dou et al., 2019) as well as in the sequencing of monoclonal antibodies (mABs) (Arndt et al., 2021). Gibson et al. demonstrated that LC‐cIMS‐MS could resolve Asp versus iso‐Asp‐containing peptides and concurrently identify the location of these modifications (Gibson et al., 2022). Lastly, LC‐cIMS‐MS was used for real‐time kinetics monitoring in the acylation of diclofenac acyl glucuronide (Higton et al., 2021).

#### 2.3.2 Coupling gas chromatography with high‐resolution ion mobility separations

Two recent works from the Jobst group used GC‐cIMS‐MS to characterize indoor dust. MacNeil et al. characterized unknown PFAS in indoor dust from their retention times and CCS. They then utilized theoretical modeling and machine learning to predict CCS based on a molecule's simplified molecular input line entry system (SMILES) to identify unknown PFAS (MacNeil et al., 2022). Breen et al. developed an algorithm to calculate the number of cycles an ion has completed in GC‐cIMS‐MS separations, which enabled higher resolution measurements of PFAS in indoor dust (Breen et al., 2022).

#### 2.3.3 Integrating mass spectrometry imaging (MSI) with ion mobility separations

**Figure 8:**
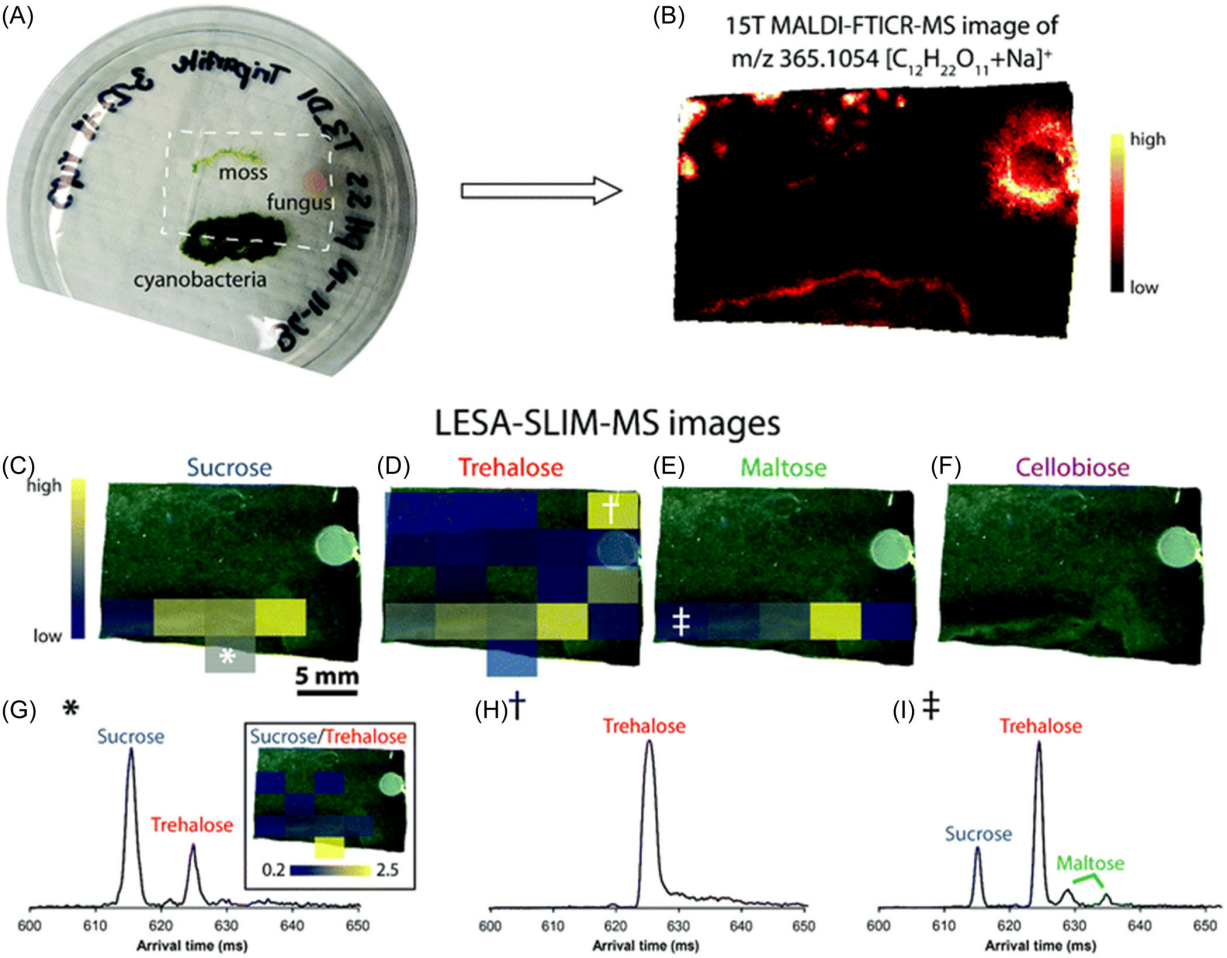
Liquid extraction surface analysis‐structures for lossless ion manipulations serpentine ultralong path with extended routing ion mobility spectrometry‐mass spectrometry provides unambiguous spatial assignment of disaccharides present in a tripartite sample consisting of moss, fungi, and cyanobacteria (A). 15 Tesla MALDI FTICR‐MS imaging (B). LESA‐SLIM‐MS images (C‐F) with IMS traces (G‐I). Reprinted (adapted) with permission from Nagy G, Veličković D, Chu RK, Carrell AA, Weston DJ, Ibrahim YM, Anderton CR, Smith RD. 2019. Towards resolving the spatial metabolome with unambiguous molecular annotations in complex biological systems by coupling mass spectrometry imaging with structures for lossless ion manipulations. Chem Commun. 55:306–309. Copyright 2019 Royal Society of Chemistry. [Color figure can be viewed at wileyonlinelibrary.com]

By integrating MSI with HRTWIMS separations, an added dimension of spatial information can be obtained. The cIMS‐MS platform can be coupled to a modular desorption electrospray ionization (DESI) source as the imaging modality, which enables an ion mobility separation dimension to be added to MSI applications (i.e., spatial annotations of compounds that cannot be resolved in the MS dimension, alone). One recent study used DESI‐cIMS‐MS to separate glycerophospholipid isomers in mouse brain (Qian et al., 2023). Quian et al. primarily used the cIMS‐MS to filter ions based on their arrival times, and when combined with CID, the data could be deconvoluted to reconstruct the precursor ions. Another recent effort from Sisley et al. used the liquid extraction surface analysis (LESA) imaging modality to extract proteins from pooled mouse brain and rat kidney tissue extracts for analysis with cIMS‐MS. They examined how many passes enabled them to identify different numbers of proteins within a selected *m*/*z* range (Sisley et al., 2020). LESA has also been coupled to SLIM IMS‐MS to provide unambiguous spatial assignment of various carbohydrate isomers in peat moss, cyanobacteria, and fungi (Figure 8) (Nagy, Veličković, et al., 2019).

### 2.4 Coupling cryogenic infrared spectroscopy with SLIM IMS‐MS

**Figure 9:**
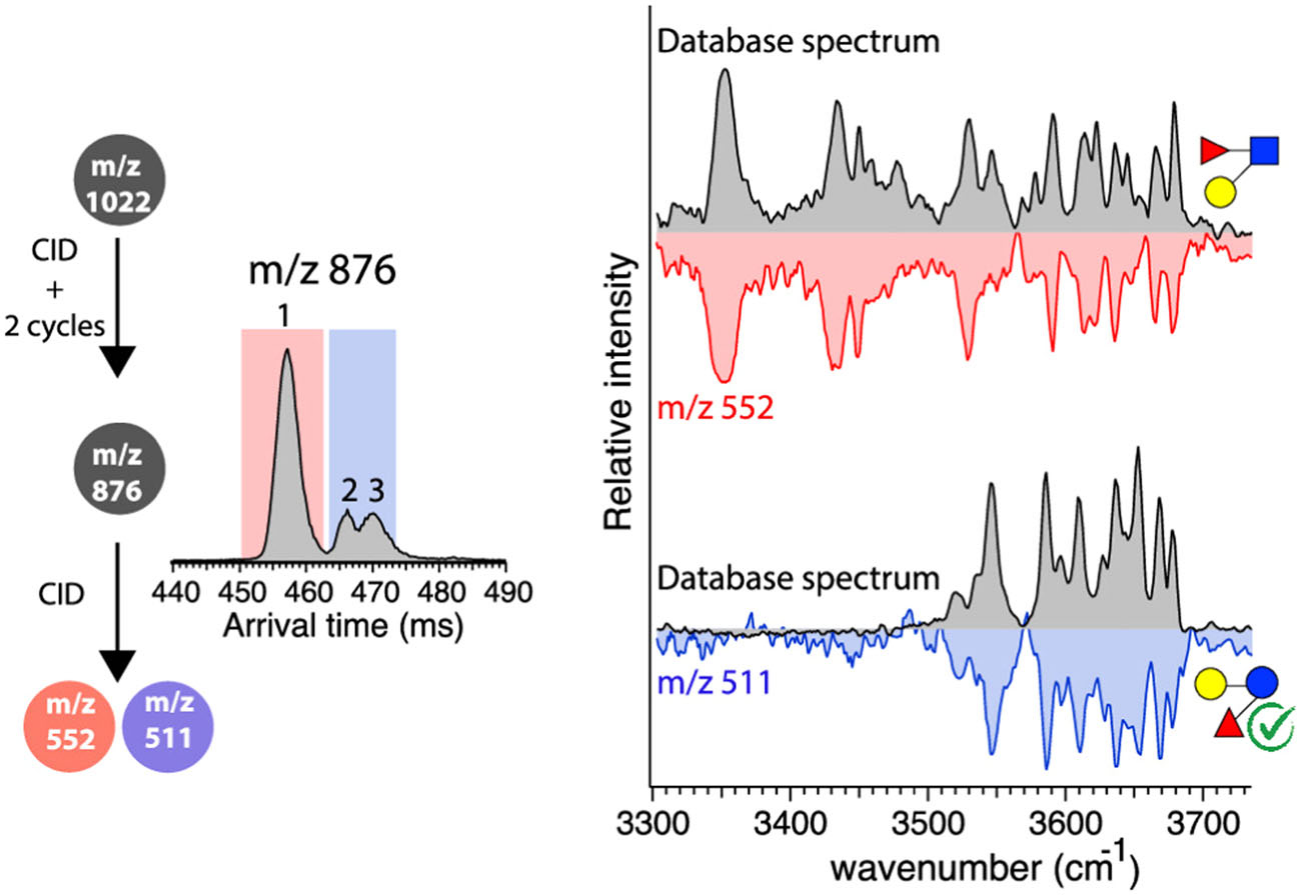
Coupling of cryo‐IR with structures for lossless ion manipulations ion mobility spectrometry‐mass spectrometry for carbohydrate sequencing. Reprinted (adapted) with permission from Bansal P, Ben Faleh A, Warnke S, Rizzo TR. 2023. Multistage ion mobility spectrometry combined with infrared spectroscopy for glycan analysis. J Am Soc Mass Spectrom. 34:695–700. Copyright 2023 American Chemical Society. [Color figure can be viewed at wileyonlinelibrary.com]

The Rizzo group has pioneered the coupling of cryogenic infrared spectroscopy to IMS‐MS separations (Bansal et al., 2020; Ben Faleh et al., 2019; Warnke et al., 2019; Yalovenko et al., 2020). Specifically, they have developed a SLIM SUPER IMS‐MS cryogenic IR spectroscopy platform that has focused on developing a database of IR fingerprints to aid in the identification of challenging isomers, with a specific emphasis on glycans (Abikhodr et al., 2021; Bansal et al., 2022; Dyukova et al., 2021; Warnke et al., 2019; Yalovenko et al., 2020). However, this approach has largely relied on authentic standards for glycan isomers, thus limiting its broad utility. To overcome this limitation, Rizzo's group developed a multistage IMS^n^ platform coupled to cryogenic IR spectroscopy (Abikhodr et al., 2023; Bansal et al., 2023; Ben Faleh et al., 2022, 2023; Pellegrinelli et al., 2022). In this newer platform, precursor glycan ions can be fragmented, and those fragment ions can be subjected to cryogenic IR spectroscopy. This can be performed in multiple stages until one of the fragment ions matches the IR fingerprint in their database. This approach effectively overcomes the reliance for authentic precursor glycan standards to be present so long as glycan fragments exist and match to their database. A general workflow for this method is illustrated in Figure 9 where lacto‐N‐difucohexaose II is fragmented, and the SLIM‐IMS separation and cryo‐IR spectra for the fragment ions are used to characterize the structure of the precursor ion. One final improvement was implementing Hadamard multiplexing to increase ion throughput (Yatsyna et al., 2022, 2023). Future work in this area will primarily focus on improving the throughput of the cryogenic IR spectroscopic measurements to enable online coupling to chromatographic separations (Abikhodr et al., 2024).

### 2.5 Macromolecules

#### 2.5.1 mABs & oligonucleotides

Several articles have explored the analysis of mABs with HRTWIMS. Interconverting conformers of IgG4 were resolved for the first‐time with cIMS‐MS (Deslignière et al., 2021, 2022). In a later effort by Deslingière et al., collision‐induced unfolding was performed on intact mABs, IgG2, IgG4, and IgG1, which gave up to 85% confidence on differentiation between subclasses (Deslignière et al., 2023). SLIM IMS‐MS was also used to characterize drug load on the light and heavy chains of a mAB (Nagy et al., 2020). Four distinct oligonucleotide isomers were also separated using cIMS‐MS, where each oligonucleotide isomer was shown to have 4‐5 conformers with their dissociation pathways and thermodynamics thoroughly characterized (Wan et al., 2023).

#### 2.5.2 Proteins/peptides

**Figure 10:**
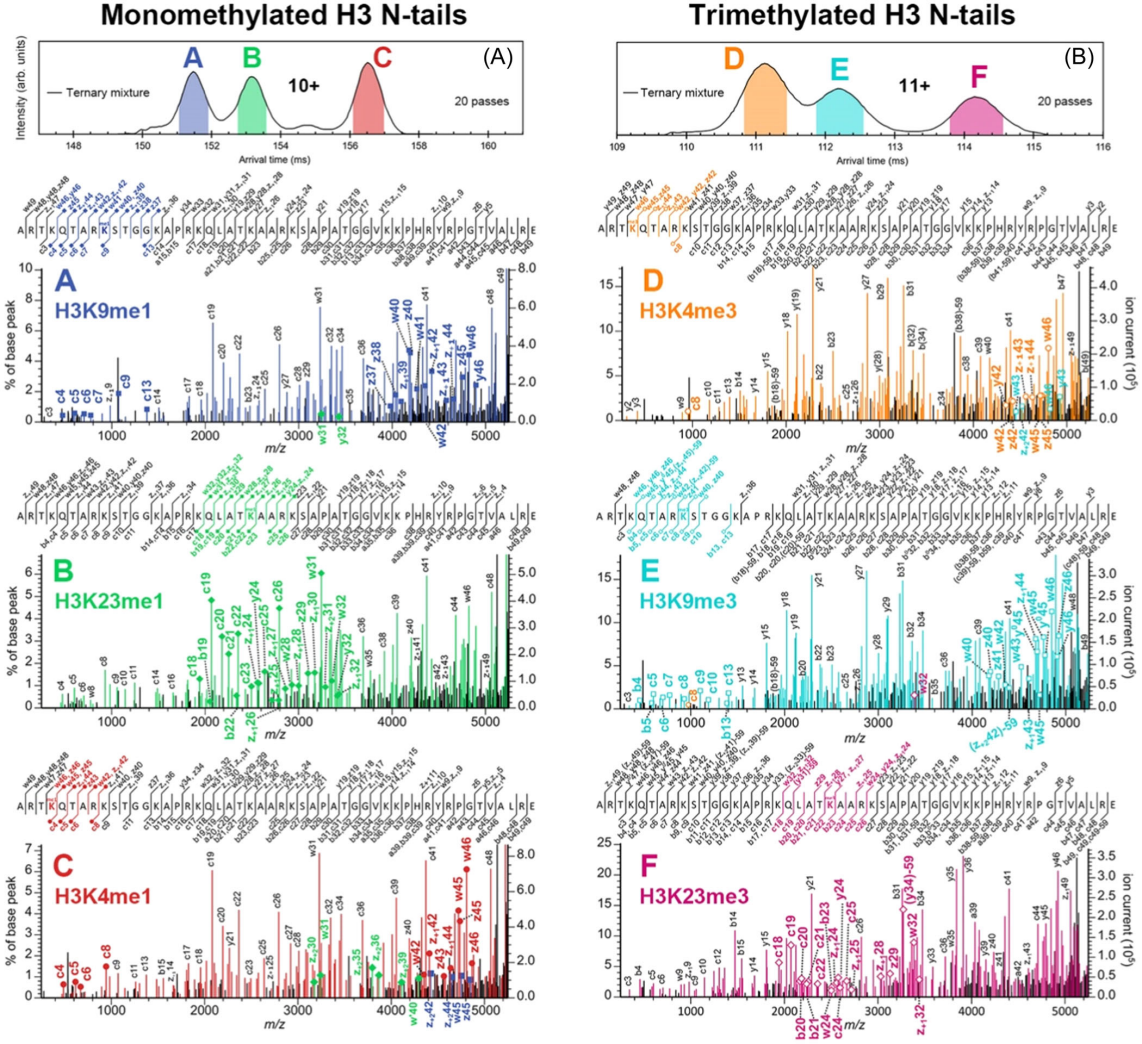
Separation and subsequent sequencing of monomethylated (A) and trimethylated (B) histone H3 N‐tail isomers with cyclic ion mobility spectrometry‐mass spectrometry/MS. Reprinted (adapted) with permission from Berthias F, Cooper‐Shepherd DA, Holck FHV, Langridge JI, Jensen ON. 2023. Full separation and sequencing of isomeric proteoforms in the middle‐down mass range using cyclic ion mobility and electron capture dissociation. Anal Chem. 95:11141–11148. Copyright 2023 American Chemical Society. [Color figure can be viewed at wileyonlinelibrary.com]

One of the fastest‐growing areas involving IMS‐MS has been in proteomics‐based workflows. Through the careful optimization of experimental parameters, the native‐like structures of cytochrome *C*, convavidin A, hIAPA, and β‐lactoglobin were retained in cIMS‐MS separations (Eldrid et al., 2019, 2021; Harrison et al., 2022). Other recent studies have focused on improving the separation of isomers and conformers, such as for hemoglobin (Sharon et al., 2023), methylated histone H3 N‐tails illustrated in Figure 10 (Berthias et al., 2023), tau phosphotripeptides from Alzheimer's disease brain tissue (Kováč et al., 2023), and β‐amyloid peptide epimers illustrated in Figure 3 (Nagy, Kedia, et al., 2019).

#### 2.5.3 Petroleomics

Cho et al. used cIMS‐MS as an ion filter to deconvolute their MS/MS spectra based on pre‐IMS quadrupole isolation and arrival time windows to improve the identification of compounds present in crude oil (Cho et al., 2019, 2021). Rüger et al. used multipass cIMS‐MS separations to better remove isobaric interferences in the convoluted m/z dimension, which improved their petroleomics analyses (Rüger et al., 2021).

### 2.6 Isomeric separations

#### 2.6.1 Glycans

**Figure 11:**
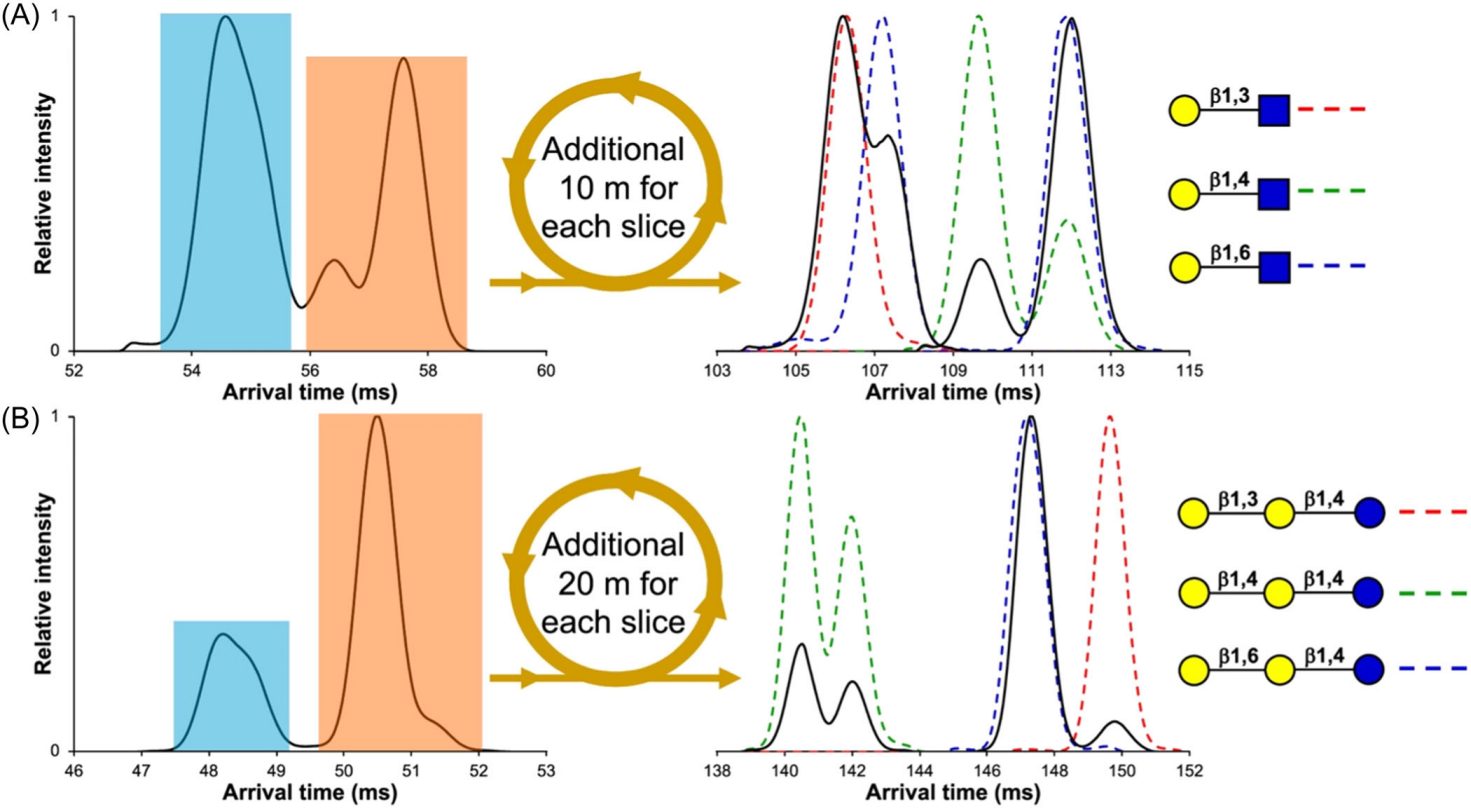
Separation of various human milk oligosaccharide isomers, including disaccharides (A) and trisaccharides (B), with high‐resolution cyclic ion mobility spectrometry‐mass spectrometry. Reprinted (adapted) with permission from Peterson TL, Nagy G. 2021a. Toward sequencing the human milk glycome: high‐resolution cyclic ion mobility separations of core human milk oligosaccharide building blocks. Anal Chem. 93:9397–9407. Copyright 2021 American Chemical Society. [Color figure can be viewed at wileyonlinelibrary.com]

Glycan‐based isomers are notoriously difficult to separate with condensed‐phase methods because of their high degree of isomeric heterogeneity stemming from their many possible carbohydrate subunits, α/β anomericities, and linkage positions. For these reasons, HRTWIMS shines as an orthogonal method to supplement traditional glycomics workflows (Figure 11).

Examples of glycan separations with HRTWIMS include both labeled and unlabeled oligosaccharide isomers, as well as their α/β anomers (Peterson & Nagy, 2021a; Ujma et al., 2019). Even with high‐resolution IMS‐MS, glycan isomers sometimes do not separate even at extended pathlengths, and thus, other approaches are needed. To this end, metal adduction, derivatization, and host–guest chemistry, are alternative strategies that have been used to resolve their isomers and anomers (Habibi & Nagy, 2023b; McKenna et al., 2019; Williamson et al., 2021). An additional challenge of characterizing glycans is the lack of authentic standards. Thus, the use of pre‐IMS‐MS/MS through CID has been implemented to fragment larger polysaccharides and measure the arrival times of the fragments to identify the possible isomers (Ollivier et al., 2023; Peterson & Nagy, 2021b). Another study implemented a molecular network to identify trends in CCS and used their molecular fingerprints to classify unknown polysaccharides (Ollivier et al., 2021). Other studies have focused on the characterization of larger polysaccharides, such as in the analysis of laminarin, dextrin, and dextran in three different species of mushrooms (Chi et al., 2022) as well as the identification of oligoporphryin isomers from red algae extracts (Ropartz et al., 2019).

#### 2.6.2 Lipids

Poad et al. used cIMS‐MS to separate double‐bond positional isomers for multiple lipid classes (e.g., fatty acids, lysophosphatidylcholine, phosphatidylcholine [PC], and phosphoinositides) to help characterize six isomers of PC 34:1 in prostate cancer cell line extract (Poad et al., 2023). For glycolipids, additional isomerization is possible at the glycan head group. Wormwood‐Moser et al. baseline separated two ganglioside isomers, ganglioside GD1a, and ganglioside GD1b, found in mouse brain extract with SLIM IMS‐MS (Wormwood Moser et al., 2021). For smaller glycosphingolipids, techniques such as permethylation and metal adduction were required to separate their isomers with cIMS‐MS (Naylor & Nagy, 2023).

#### 2.6.3 Small molecules

**Figure 12:**
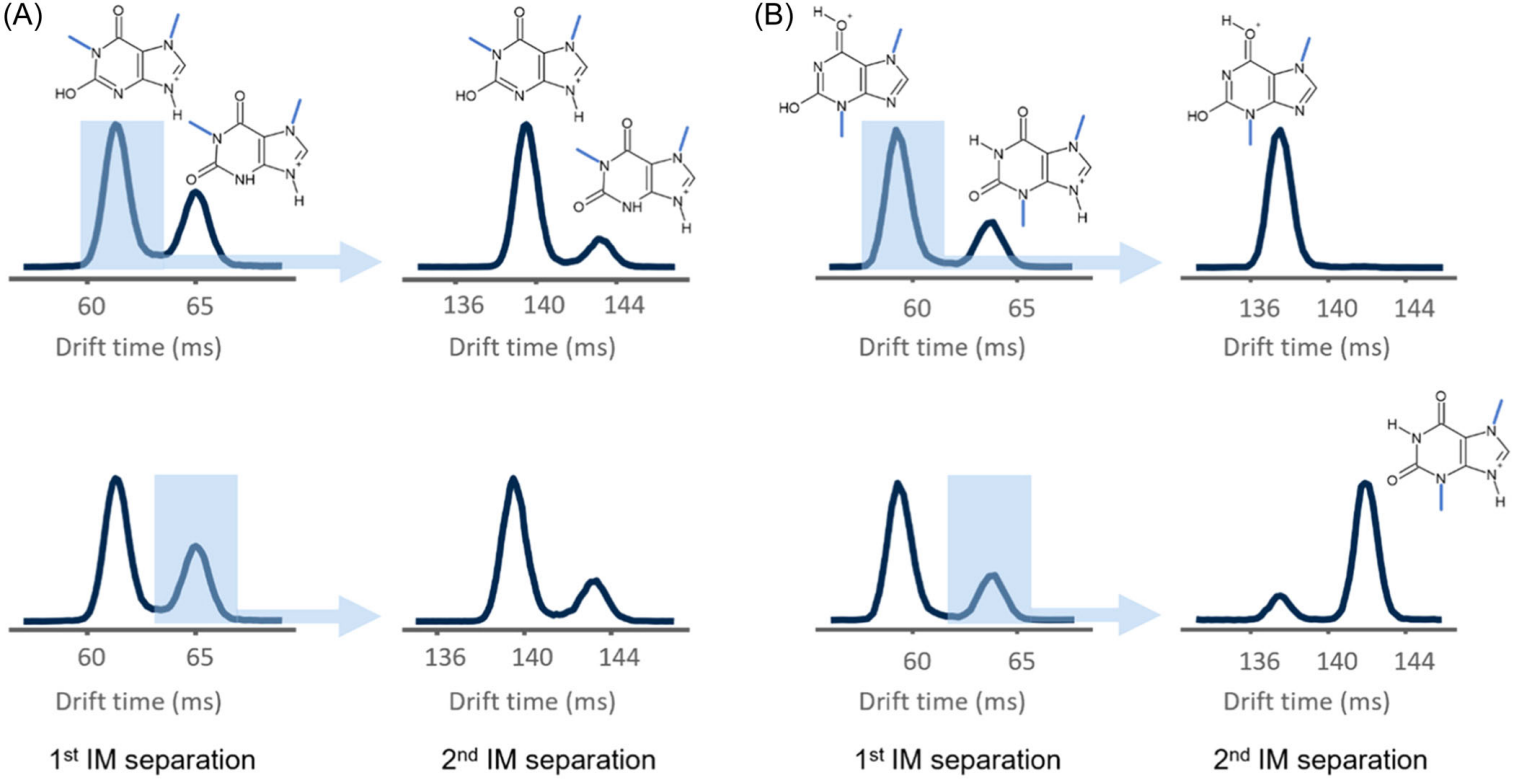
Separation of the isomers, protomers, and tautomers of various caffeine‐based metabolites, including paraxanthine (A) and theobromine (B), with cyclic ion mobility spectrometry‐mass spectrometry. Reprinted (adapted) with permission from Sepman H, Tshepelevitsh S, Hupatz H, Kruve A. 2022. Protomer formation can aid the structural identification of caffeine metabolites. Anal Chem. 94:10601–10609. Copyright 2022 American Chemical Society. [Color figure can be viewed at wileyonlinelibrary.com]

Other notable separations include those of small molecules, such as posttranscriptionally modified isomers of RNA (methylcystine and methyladiosine) as well as uridine and pseudo‐uridine isomers in a total RNA extract with cIMS‐MS (Kenderdine et al., 2020). Through dimerization, R/S‐propanolol and R/S‐thalidomide were resolved with cIMS‐MS, which enabled high‐accuracy purity measurements (Cooper‐Shepherd et al., 2022). Catechin epimers and other flavonoid isomers present in tea extracts were separated with cIMS‐MS, thus demonstrating potential for real‐time monitoring of these compounds (de Bruin et al., 2023). Finally, highlighted in Figure 12, the structural isomers, protomers, and tautomers of various caffeine‐based metabolites could be resolved with cIMS‐MS (Sepman et al., 2022). Future work in this area may include more efforts related to the separation of the isomers of illicit substances, including synthetic cannabinoids (Aderorho et al., 2024).

## 3 FUTURE PROSPECTS

With the growing use of high‐resolution traveling wave ion mobility separations each year, the prospects of this technique look bright. New SLIM IMS‐MS techniques and instrumentation are constantly being developed, largely due to the flexibility of board designs and open‐source availability. Specifically, we anticipate more efforts related to the coupling of SLIM IMS to high‐resolution Orbitrap‐based mass analyzers. While the cIMS‐MS platform is commercially available, there is tremendous flexibility afforded by the cIMS array, such as enabling multiple stages of IMS^n^, peak slicing experiments, and combinations of IMS and MS/MS. In our view, IMS‐MS will become increasingly incorporated with other techniques such as MSI, chromatographic separations, and cryo‐IR. These instrument platforms and related applications will enable the next generation of omics‐based analyses.

## AUTHOR CONTRIBUTIONS

**Cameron N. Naylor**: Conceptualization; investigation; writing—original draft; writing—review/editing. **Gabe Nagy**: Conceptualization; writing—original draft; writing—review/editing.

## CONFLICT OF INTEREST STATEMENT

The authors declare no conflict of interest.
